# Physiological, Pathological and Pharmacological Interactions of Hydrogen Sulphide and Nitric Oxide in the Myocardium of Rats with Left Ventricular Hypertrophy

**DOI:** 10.3390/cimb44010030

**Published:** 2022-01-16

**Authors:** Ashfaq Ahmad

**Affiliations:** Department of Pharmacy practice, College of Pharmacy, University of Hafr Al-Batin, Hafr Al-Batin 31991, Saudi Arabia; ashfaqa@uhb.edu.sa; Tel.: +966-504309874

**Keywords:** hydrogen sulphide, nitric oxide, left ventricular hypertrophy, cystathione γ lyase (CSE) endothelial nitric oxide synthase (eNOS)

## Abstract

Left ventricular hypertrophy (LVH) is characterized by increased myocardium thickness due to increased oxidative stress and downregulation of cystathione γ lyase (CSE) endothelial nitric oxide synthase (eNOS). Upregulation of CSE by hydrogen sulphide (H_2_S) and ENOS by L-arginine can arrest the progression of LVH individually. The present study explored the combined treatment of H_2_S and NO in the progression of LVH, and demonstrated that the response is due to H_2_S, NO or formation of either new molecule in physiological, pathological, and pharmacological in vivo settings of LVH. Exogenous administration H_2_S+NO in LVH significantly reduced (all *p* < 0.05) systolic blood pressure (SBP) and mean arterial pressure (MAP), LV index, heart index and oxidative stress when compared to the LVH group. There was downregulation of CSE mRNA and eNOS in the heart, and exogenous administration of H_2_S+NO groups upregulated eNOS MRNA while CSE MRNA remained downregulated in the hearts of the LVH group. Similar trends were observed with concentrations of H_2_S and NO in the plasma and tissue. It can be concluded that combined treatment of LVH with H_2_S and NO significantly ameliorate the progression of LVH by attenuating systemic hemodynamic and physical indices, and by decreasing oxidative stress. Molecular expression data in the myocardium of LVH depicts that combined treatment upregulated eNOS/NO while it downregulated CSE/H_2_S pathways in in vivo settings, and it is always eNOS/NO pathways which play a major role.

## 1. Introduction

The family of endogenous gaseous mediators comprised of nitric oxide (NO), hydrogen sulphide (H_2_S) and carbon monoxide (CO) [1]. NO is produced from three isoforms of nitric oxide synthase (NOS); endothelial nitric oxide synthase (eNOS), inducible nitric oxide synthase (iNOS), and neuronal nitric oxide synthase (nNOS) [2]. Endothelial nitric oxide synthase (eNOS) is constituted in left ventricular myocytes [3,4]. Literature reported that upregulation of eNOS in the myocardium arrests the progression of left ventricular hypertrophy (LVH) [4]**,** and activation of eNOS in cardiomyocytes is a well-known antihypertrophic agent [5]. NO has earned its repute in cardiovascular systems as a potent vasodilator [6]. Therapeutic effects of NO in LVH are multifactorial, and lowering of systemic hemodynamics is one of the factors [4,7]. It can be deduced from the above literature that provision of the ENOS/NO pathway arrests the progression of LVH, while on the other side, deficiency of eNOS can develop sever cardiac hypertrophy [8]. Although the role of NO in the hypertensive model of LVH has been studied previously [9,10], few studies explored whether the antihypertrophic role of NO is dependent or independent of the lowering of blood pressure. It was interesting to know that supplementation of L-arginine, an NO donor, played an antihypertrophic role in the genetic model of hypertensive rats without lowering of blood pressure [11]; this same role is attributed with the lowering of blood pressure in salt-sensitive spontaneously hypertensive rats (SHR) [12]. It can be concluded from the above arguments that NO plays an antihypertrophic role by upregulating the eNOS/NO pathway, lowering blood pressure, and ameliorating oxidative stress.

The second member of this gaseous transmitter family is H_2_S which is produced by three enzymes: cystathione γ lyase (CSE), particularly in the heart [1,4]; cystathione β synthase (CBS), especially in the kidney [13,14]; and 3-mercaptopyruvate sulphur transferase (MST), in the brain [15]. H_2_S, having the advantage of being produced in the heart, arrests the progression of LVH by upregulation of the CSE/H_2_S pathway in the myocardium [4] and cardiac hypertrophy in the abdominal aortic coarctation model in Sprague-Dawlwy rats [16]. Among the possible mechanisms involved is the antihypertrophic effect of lowering of blood pressure [17] by modulating vascular tone [18]. Another significant factor in the pathogenesis of LVH is oxidative stress in cardiac remodeling [19,20]. It has been reported that upregulation of CSE/H_2_S pathways arrest the progression of LVH by modulating oxidative stress [21]. Increased oxidative stress result in endothelial dysfunction by increasing pulse wave velocity, and exogenous treatment with antioxidants like vitamin C and H_2_S have been found to reverse the damage done by oxidative stress in essential hypertension [22] and in LVH [21]. 

Interaction of H_2_S and NO produces a crosstalk that H_2_S either acts at the level of NOS, nitric oxide influences CSE activity, or that H_2_S (or species derived from it at physiological pH) interact with vascular-derived nitric oxide and vice versa with apparent formation of a ‘nitrosothiol’-like species, summarized in the reported literature [23]. We previously reported that L-arginine, a donor of NO, increases the H_2_S concentration in normal animals, but this interaction was absent in the LVH disease model when treated with an NO donor [24]. In contrast, exogenous administration of H_2_S increased the NO concentration in the plasma in both normal animals and in the LVH diseased model, which suggests a positive interaction exists between H_2_S and NO [14]. The present study was designed to explore the interaction of exogenous administrations of H_2_S and NO donors simultaneously in the progression of LVH. Furthermore, study was also designed to explore the interactions between H_2_S and NO when donors of both gaseous transmitters were given together in in vivo settings of physiological, pathological, and pharmacological conditions.

## 2. Materials and Methods

### 2.1. Study Groups

All the male *Wistar-Kyoto* (*WKY*) rats weighing 200–220 g, and of 8–9 weeks of age, were procured from the Animal Research and Service Centre (ARASC) of Universiti Sains Malaysia and kept for acclamitization for 5 days in the transit room facility of the School of Pharmaceutical Sciences, Universiti Sains Malaysia. All the animals were given free access to tap water and standard chow food (Gold Coin Sdn. Bhd., Port Klang, Penang, Malaysia). Experimental procedure for the execution of experiments was approved by the Animal Ethical Committee of Universiti Sains Malaysia, via approval letter number USM/Animal Ethic Approval/2012/(76)/(364), dated 14 March 2012.

### 2.2. Induction of Left Ventricular Hypertrophy (LVH)

Left ventricular hypertrophy (LVH) was induced in WKY rats by using some modification as reported [25]. In this model, isoprenaline (β-adrenergic agonist) was administered subcutaneously on days 1, 3, 7, 10 and 13 (72 h apart), while caffeine was given in drinking water 62 mg/L every day for 14 days. This approach was a modification of the previously reported model for LVH [26] by using 4 sub-cutaneous injections of isoprenaline (72 h apart) and 62 mg/L oral administration of caffeine for 14 days.

### 2.3. Experimental Groups

Animals were randomly divided into 8 groups (4 groups for in vivo (n = 6) and 4 groups for molecular study (3 animals in each group and each having a triplicate, so in total 3 × 3 = 9 in one group)) consisting of (1) Control WKY, (2) LVH-WKY, (3) Control-H_2_S+NO, and (4) LVH-H_2_S+NO. These groups were designed to achieve the objective laid in this study. Main groups were designed to study the impact of H_2_S, NO alone, and the combination of H_2_S+NO in the regression of LVH combined treatment by exogenous hydrosulfide (NaHS, donor of H_2_S) plus nitric oxide (L-arginine, donor of NO) treatment.

Selected groups of experimental animals were treated with combined administration of sodium hydrosulfide at a dose of 56 µmol/kg of NaHS intraperitoneally each day for 5 weeks [17]**,** and L-arginine at a dose of 1.25% in drinking water for 5 weeks [27] was administered to evaluate the combined effect of exogenous H_2_S+NO in the attenuation of LVH. 

### 2.4. Electrocardiogram (ECG) Recording in Anaesthetized Rats

Electrocardiogram (ECG) was done by using lead II on day 35 as reported [25]. Animals were fasted overnight before being anaesthetized with 60 mg/kg i.p. injection of sodium pentobarbitone (Nembutal, CEVA, Marseille, France). After complete induction of anaesthesia, a standard 3-lead (left foreleg, right foreleg, and left rear leg) surface ECG recording of six LVH or control rats was performed as previously described using gold-plated needle electrodes (ADInstruments, Sydney, Australia) inserted under the skin [25,28,29]. Recordings were made within 2–3 min using a differential amplifier attached to a PowerLab data acquisition system (PowerLab, ADInstruments, Australia) and averaged for each rat. Data were then combined from the averaged measurements for each individual rat to yield an averaged value for each group. ECG was done as mentioned earlier. However, R-R intervals, R-amplitude, QRS complex interval, QT interval and QTc were included to analyze the LVH on day 35, which is the terminal day of experiment, and the final day ECG data was given high priority for analysis.

### 2.5. Preparation and Surgical Procedure for Acute Experiment 

In vivo renal vasoconstrictor responses studies were performed as previously reported [30]. Briefly, after the onset of anaesthesia by using 60 mg/kg i.p injection of sodium pentobarbitone (Nembutal, CEVA, Marseille, France) and ECG recording, the trachea was exposed by a midline incision at the neck region and cannulated by PP240 tubing (Portex, Hive, Kent, UK) for proper ventilation during the experiment.

After tracheotomy, the carotid artery was cannulated by PP50 tubing (Portex, Hive, Kent, UK) and connected to a pressure transducer (model P23 ID Gould, Statham Instruments, UK) which was further attached to a PowerLabdata acquisition system (PowerLab, ADInstruments, Sydney, Australia) for monitoring of mean arterial blood pressure (MAP) and heart rate (HR). The jugular vein was also cannulated by PP50 tubing (Portex, Kent, UK) for infusion of saline and a maintenance dose of anaesthesia when required. The left kidney and aorta were then exposed by a mid-line abdominal incision and a laser Doppler probe (ADInstruments, Sydney, Australia) was placed at the cortical position of the kidney to measure renal cortical blood perfusion (RCBP). The bladder was cannulated to ease the gravitational flow of the urine and facilitate the excretion of drugs via urine during the three-phase experiment. The left iliac artery was then cannulated and the animal was left to stabilize for 1 h to record the base line values of systemic hemodynamics like SBP, DBP, MAP, HR, PP and time for pulse wave velocity (PWV).

### 2.6. Measurement of Heart and Left Ventricle Indices

The extent of hypertrophy was evaluated by removing the heart from the body at the end of experiment and carefully weighing it. Left ventricle was carefully isolated from heart. The thickness of the myocardium and LV chamber internal diameter was estimated as described previously [31]. Thickness was measured with a Vernier calliper just below the level of the mitral valve. These values were used to generate heart and LV indices as follows:

Heart Index = Heart weight/body weight × 100

Left ventricle Index = Left ventricle weight/body weight × 100

### 2.7. Measurement of Antioxidant Assays in the Plasma

Plasma levels superoxide dismutase activity (SOD), malondialdehyde (MDA), glutathione reductase (GSH), total antioxidant capacity (T-AOC) and nitric oxide activity (NO) were measured in plasma and in heart tissue using laboratory kits (NJJC Bio Inc., Nanjing, China) following the instruction provided by the manufacturer.

### 2.8. Determination of Concentration of H_2_S and Nitric Oxide in the Plasma and H_2_S in the Urine 

Concentration determination of H_2_S and NO was done as reported in detail [4].

### 2.9. Quantification of Cystathione γ Lyase mRNA and Endothelial Nitric Oxide Synthase mRNA in the Myocardium Using RT-PCR

Quantification of CSE and ENOS MRNAS were done as reported in detail [4]. However, TaqMan primers and probes (TaqMan^®^-Gene Expression assays, Applied Biosystems, Carlsbad, CA, USA) were as follows:

1. CSE (Gen Bank accession No. NM_017074.1 and Rn00567128_m1) gene [4].
2. eNOS (Gen Bank accession No. NM_021838.2 and Rn02132634_s1) gene [32]
3. β-actin (Gen Bank accession No. NM_031144.2 and Rn00667869_m1) gene [33].

The following primers for CSE, eNOS, and internal control beta-actin along with TaqMan chemistry were used (assay ID: Rn00567128_ml, Rn002132634_s1 and Rn00667869_ml, respectively) for the gene expression assay.

### 2.10. Histopathology Study of the Left Ventricle Using PicroSirus Red, Haematoxylin and Eosin (H&E) Staining

The left ventricles (LVs) of all rats were collected after careful isolation from heart. The LV tissue was blotted dry on a filter paper and kept in 10% formalin solution for preservation. After the subsequent steps of embedding, trimming and sectioning, the LV tissue underwent staining with haematoxylin and eosin as reported previously [4].

### 2.11. Statistical Analysis 

The statistical analysis was performed using a one-way analysis of variance followed by a Bonferroni post hoc test using GraphPad Prism software (GraphPad Software, San Diego, CA, USA), while gene expression data was analysed using the comparative method (∆∆CT method) and using the StepOne™ Software (Version 2.1, Applied Biosystem, Carlsbad, CA, USA). All data were presented as mean ± S.E.M. with significance at *p* < 0.05.

## 3. Results

### 3.1. Electrocardiogram (ECG) Recording in Anaesthetized Rats

The R-R interval (duration of cardiac cycle), R amplitude (ventricular stimulation) and QRS (ventricular depolarization) interval of all the four experimental groups, i.e., Control-WKY, Control-H_2_S+NO LVH-WKY and LVH-H_2_S+NO groups, showed significant changes in the baseline of the R-R interval, R amplitude and QRS interval at day 35. The R-R interval, R amplitude and QRS interval of LVH-WKY was significantly greater (*p* < 0.05) when compared to Control WKY (all *p* < 0.05), while LVH-H_2_S+NO showed significantly lower (*p* < 0.05) R-R interval, R amplitude and QRS interval when compared to that of LVH-WKY on day 35, as shown in Table 1. The QT interval of LVH-WKY was significantly greater (*p* < 0.05) when compared to Control WKY. The remaining LVH-H_2_S+NO groups showed a significantly higher (*p* < 0.05) QT interval when compared to Control WKY on day 35, as shown in Table 1.

### 3.2. Measurement of Systemic Hemodynamics in Anaesthetized Rats

**Figure 1:**
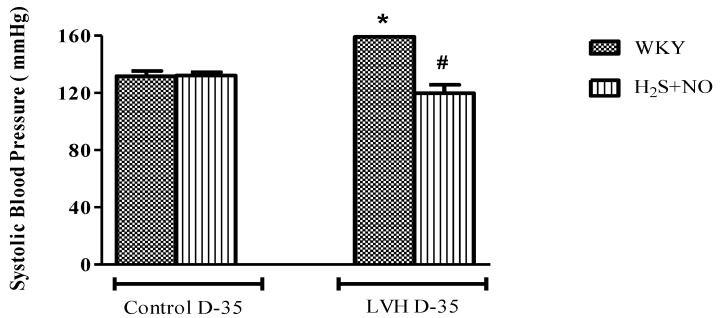
(**A**,**B**). The changes in systolic blood pressure (SBP) and mean arterial pressure (MAP) of WKY, and H_2_S+NO groups of Control and LVH groups taken during acute experiment on day 35. Data is presented as mean ± SEM (n = 6), *p* < 0.05. Statistical analysis was done by one-way analysis of variance (ANOVA) followed by Bonferroni post hoc tests for all the groups on day 35. * vs. Control WKY, # vs. LVH-WKY.

Systolic blood pressure (SBP) and mean arterial pressure (MAP) of all the four experimental groups, i.e., Control-WKY, Control-H_2_S+NO, LVH-WKY and LVH-H_2_S+NO groups, showed significant changes in baseline SBP and MAP on day 35. However, there was a significant increase (*p* < 0.05) in SBP and MAP in LVH-WKY when compared to Control WKY, while the remaining LVH-H_2_S+NO showed a significantly lower (*p* < 0.05) SBP and MAP when compared to that of LVH-WKY on day 35, as shown in Figure 1A,B.

### 3.3. Measurement of LV Index and LV Internal Diameter

**Figure 2:**
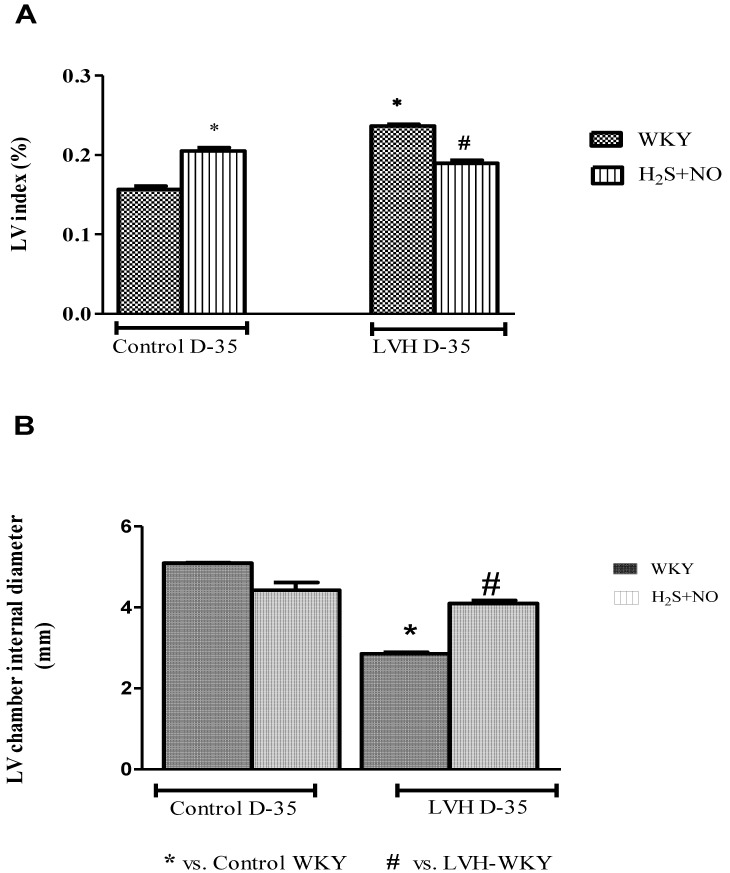
The changes in LV indices (**A**) and LV internal diameter (**B**) of WKY and H_2_S+NO groups of Control and LVH groups taken at the end of acute experiment on day 35. Data is presented as mean ± SEM (n = 6), *p* < 0.05. Statistical analysis was done by one-way analysis of variance (ANOVA) followed by Bonferroni post hoc tests for all the groups on day 35.

LV index and LV internal diameter of all the four experimental groups—Control-WKY, Control-H_2_S+NO, LVH-WKY and LVH-H_2_S+NO groups—showed significant changes in baseline on day 35, as shown in Figure 2A,B. However, there was a significant increase (*p* < 0.05) in LV index and a significant decrease (*p* < 0.05) in LV internal diameter in LVH-WKY when compared to that in Control WKY, while the remaining LVH-H_2_S+NO showed a significantly lower (*p* < 0.05) LV index and a significantly greater (*p* < 0.05) LV internal diameter when compared to that of LVH-WKY on day 35, as shown in Figure 2A,B. 

### 3.4. Measurement of SOD, MDA, GSH and T-AOC in the Plasma 

**Figure 3:**
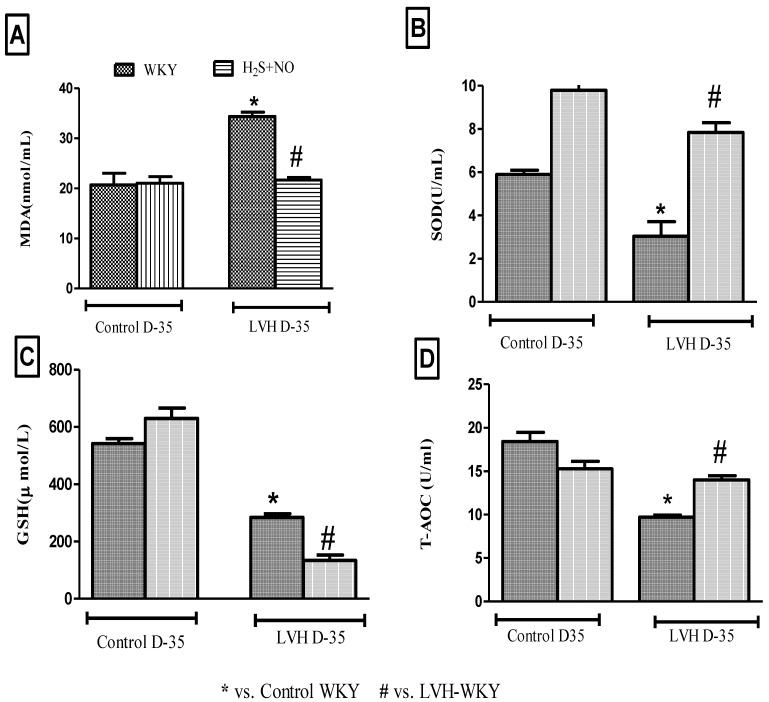
(**A**–**D**): The changes in plasma levels of MDA (**A**), SOD (**B**), GSH (**C**) and TAOC (**D**) of WKY and H_2_S+NO groups of Control and LVH groups taken on day 35. Data is presented as mean ± SEM (n = 6), *p* < 0.05. Statistical analysis was done by one-way analysis of variance (ANOVA) followed by Bonferroni post ho*c* tests for all the groups on day 35.

SOD and MDA of all the four experimental groups—Control-WKY, Control-H_2_S+NO, LVH-WKY and LVH-H_2_S+NO groups—showed significant changes in baseline on day 35 as shown in Figure 3A,B. However, there was a significant increase (*p* < 0.05) in MDA and a significant decrease (*p*<0.05) SOD in LVH-WKY when compared to that in Control WKY, while the remaining LVH-H_2_S+NO showed a significantly lower (*p* < 0.05) MDA and a significantly greater (*p* < 0.05) SOD when compared to that of LVH-WKY on day 35, as shown in Figure 3A,B. GSH and T-AOC were significantly reduced (all *p* < 0.05) in LVH-WKY, while LVH-H_2_S+NO did not show any significant change in GSH; however, T-AOC was increased significantly when compared to the LVH-WKY group, as shown in Figure 3C,D. 

### 3.5. Measurement of NO, H_2_S in the Plasma and H_2_S in the Urine

**Figure 4:**
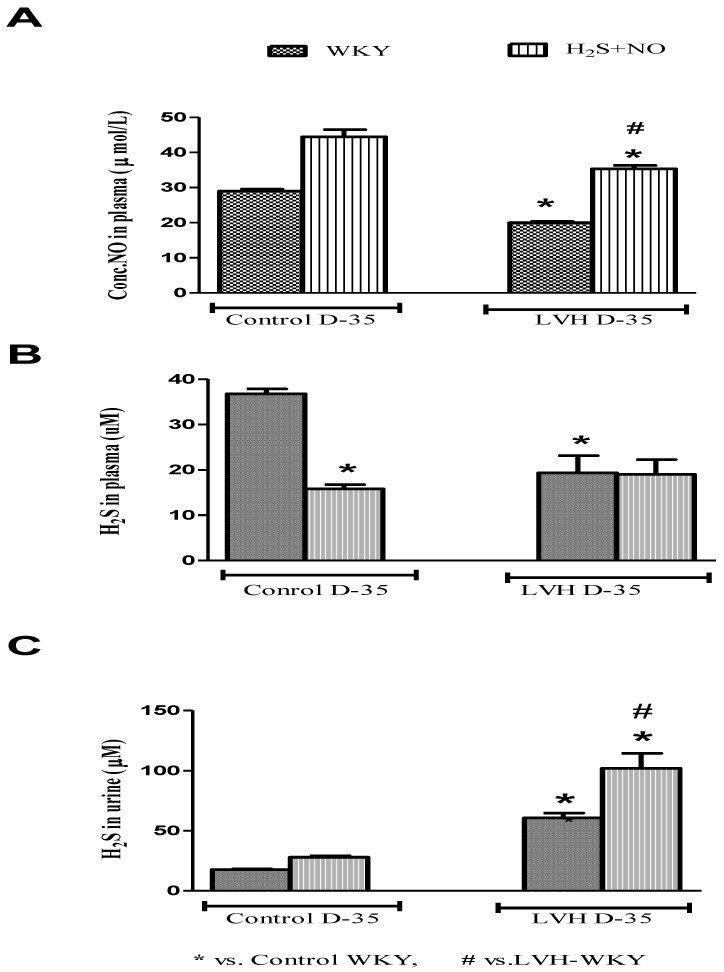
(**A**–**C**): The changes in plasma levels of NO, H_2_S and urinary H_2_S of WKY and H_2_S+NO groups of Control and LVH groups taken on day 35. Data is presented as mean ± SEM (n = 6), *p* < 0.05. Statistical analysis was done by one-way analysis of variance (ANOVA) followed by Bonferroni post hoc test for all the groups on day 35.

NO and H2S in the plasma of all the four experimental groups—Control-WKY, Control-H_2_S+NO, LVH-WKY and LVH-H_2_S+NO groups—showed significant changes in baseline on day 35, as shown in Figure 4A,B. However, there was significant decrease (all *p* < 0.05) in NO and H2S in the plasma of LVH-WKY when compared to that in Control WKY, while the remaining LVH-H_2_S+NO showed a significantly higher (*p* < 0.05) NO in the plasma and insignificant changes observed in the concentration of H_2_S in the plasma when compared to that of LVH-WKY on day 35, as shown in Figure 4A,B. It was surprising to know that induction of disease and treatment with H_2_S and NO in LVH has significantly increased (all *p* < 0.05) the excretion of H_2_S in the urine when compared to that in Control WKY, as shown in Figure 4C.

### 3.6. Quantification of CSE and ENOS MRNAS Expressions in the Heart

**Figure 5:**
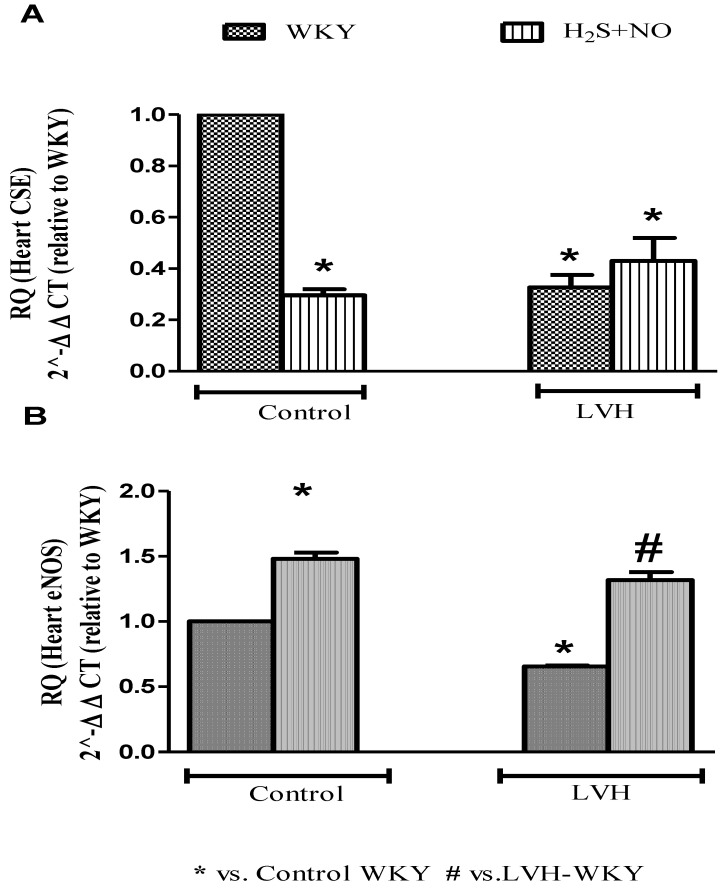
Relative quantification of heart (**A**) CSE (mRNA) and (**B**) eNOS (mRNA) of WKY and H_2_S+NO groups of Control and LVH rats. Mean fold changes (relative to WKY and normalized to β-actin) were calculated by the 2^−ΔΔCT^ method. Data expressed as mean ± SEM and difference between the mean were analysed by one-way ANOVA followed by Bonferroni post hoc test. (n = 3 in each group while each animal has triplicate sample, so experimental n = 9). * *p* < 0.05 vs. WKY and # *p* < 0.05 vs. LVH-WKY.

CSE and eNOS mRNAs expressions in the heart of all the four experimental groups—Control-WKY, Control-H_2_S+NO, LVH-WKY and LVH-H_2_S+NO groups—showed significant changes in baseline on day 35, as shown in Figure 5A,B. However, there was a significant decrease (all *p* < 0.05) in CSE and eNOS mRNAs expressions in the heart of LVH-WKY when compared to that in Control WKY, while remaining LVH-H_2_S+NO showed significantly higher (*p* < 0.05) eNOS mMRNAS expressions in the heart and insignificant changes observed in the expression of CSE mRNAs expressions in the heart when compared to that of LVH-WKY on day 35, as shown in Figure 5A,B.

### 3.7. Histopathology of Heart Tissue by Using Hematoxyllin and Eosin Staining

**Figure 6:**
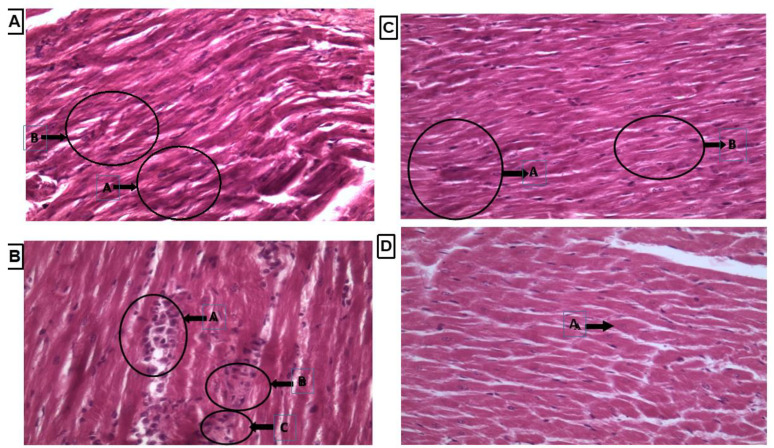
(**A**–**D**): Histopathology of heart tissues of Control WKY, LVH-WKY, Control H_2_S+NO and LVH-H_2_S+NO by using hematoxylin and eosin staining.

Heart tissue showed normal shape cardiac nuclei and muscle striation, as shown in Figure 6A, while LVH-WKY showed unevenly distributed degenerated cardiac nuclei and the presence of fibroblasts, indicating inflammation. Some evidence of fibrosis and loss of muscle striation were also observed, as shown in Figure 6B. Treatment with H_2_S+NO almost restored the shape of cardiac nuclei, without any signs of fibrosis, with few hypertrophied cells and inflammation, as shown in Figure 6C,D.

### 3.8. Histopathology of Heart Tissue by Using Picrosirius Red Stain

**Figure 7:**
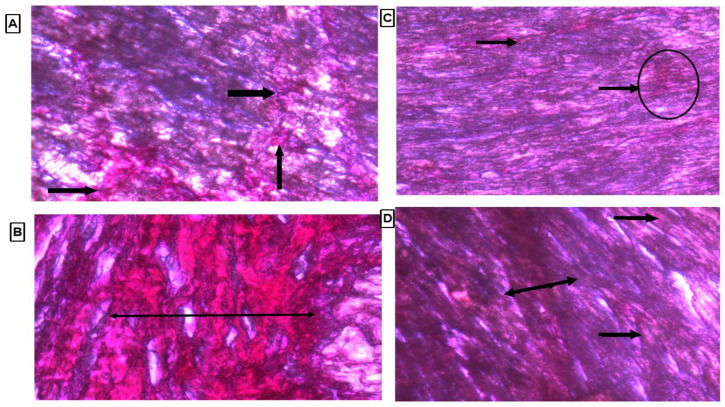
(**A**–**D**): Histopathology of heart tissues of Control WKY, LVH-WKY, Control H_2_S+NO and LVH-H_2_S+NO by using Picrosirius Red staining.

Collagen content was observed by observing the thin strands or bands of collagen stained as red color on the cardiac tissue. There were marked thick bands of collagen on cardiac tissue in the LVH-WKY group, as shown in Figure 7B. Treatment with H_2_S+NO in Control and LVH groups led to observation of few thin bands of collagen other than diffused and scattered collagen as compared to the LVH-WKY group with the naked eye, as shown in Figure 7C,D, respectively. 

## 4. Discussion

The present study was designed to explore the effect of simultaneous administrations of H_2_S and NO donors in the progression of LVH. Secondly, the current study also set an objective to understand the interaction between both gaseous transmitters H_2_S and NO when both are given together in in vivo physiological, pathological, and pharmacological settings. The present study demonstrated the novel findings that simultaneous administrations of H_2_S and NO arrest the progression of LVH by reducing systemic parameters, increasing antioxidant status and reversing the histopathological modifications in LVH. Secondly, when administered simultaneously, there is always up-regulation of the eNOS/NO pathway, while also downregulation of the CSE/H_2_S pathway in physiological and pharmacological in vivo settings. 

In the present study it was observed that the R-R interval, R-amplitude and QRS complexes were increased in the LVH-WKY group when compared to Control WKY on day 35. This would imply a slower heart rate, since ventricle must contract with a greater force to overcome the preload, and the size of the heart and ventricle was increased, and more time was required for the electrical impulses to complete the depolarization and repolarization cycle respectively on day 35 in the LVH-WKY group compared to the Control group. This model of LVH may be a transition state between LVH and heart failure. Thus, the increase in cardiac cycle duration in the LVH group is consistent with a prognosis which may progress into heart failure. These data are in line with a previous report using a similar LVH model [25]. Treatment with H_2_S+NO in the LVH group caused a reduction in the R-R interval, R-amplitude and QRS complexes when compared to the untreated LVH group. These findings indicate that there was a regression in the amount of hypertrophied cardiac tissue, which would ultimately reduce the time required to complete the cardiac cycle. The outcomes of this study have established that the pharmacological induction of LVH using the isoprenaline/caffein model meant that the R-R interval, R-amplitude, QRS complex and QT interval were increased. It should be recognized that these ECG data were obtained in the anesthetized state which may differ from those obtained in conscious rats. However, it is evident that these different parameters of ECG (R-R interval, R-amplitude, QRS complex and QT interval) are increased in this model of hypertrophy, but can be modulated by treatment with H_2_S+NO when compared to individual treatment, as shown in Table S3.

The combined treatment using H_2_S+NO reduced the SBP and MAP more effectively and significantly as compared to the individual treatments with H_2_S and NO alone, as shown in Supplementary File Table S1. No previous findings related to the effect of combined H_2_S+NO treatment on blood pressure have been reported, although our group reported individual effects of H_2_S [21] and NO [4] in the progression of LVH. However, different schools of thought have proposed different potential interactions between H_2_S and NO. One possibility is that there are individual contributions from H_2_S and NO in the combined treatment which may contribute to a synergistic effect, either in an additive or potentiating way. There are several theories which point to H_2_S and NO enhancing each other’s production [34,35,36]. Some authors suggest there is formation of an intermediate molecule (perhaps nitrosthiol), which may modulate the function of H_2_S and NO [37]. Mechanisms underlying an interaction between H_2_S and NO have not yet been clarified but the present findings represent one of the first pieces of evidence demonstrating that combined treatment with H_2_S+NO significantly reduces systemic hemodynamics and arrest the progression of LVH.

The combined treatment with H_2_S+NO in the Control and LVH groups resulted in a greater decrease in the LV index, and improved the LV internal diameter when compared to the LVH-WKY group. LVH index in the LVH-H_2_S+NO group was reduced to a significantly greater extent compared to the individual treatments with either H_2_S or NO alone, as shown in Supplementary File (Table S2). No previous study has reported the effect of a combination of H_2_S+NO on the changes in the cardiac physical indices. Modulation of physical indices may be due to the antihypertrophic role of NO [4,38] and this phenomenon can be attributed to H_2_S [21]. Thus, this is one of the first reports demonstrating that treatment with H_2_S+NO in LVH caused a greater decrease in LV index and increased the LV chamber internal diameter compared to individual treatments with H_2_S and NO, as shown in Table S2. 

The combined treatment with H_2_S+NO in LVH resulted in reduced MDA, GSH and increased SOD and T-AOC levels in the plasma, as shown in Figure 3A–D. Limited or no data are available regarding the antioxidant potential of H_2_S+NO in the LVH rats, although individual effects of H_2_S and NO on oxidative stress parameters are shown in Supplementary File Table S4. Although in combined treatment groups of Control WKY and LVH-WKY the concentration of H_2_S in plasma is reduced and concentration of NO in plasma is increased, even then the antioxidant status of LVH-H_2_S+NO was upregulated. These observations show that NO is playing a dominant role in maintaining the balance between pro-oxidants and antioxidants while the low concentration of H_2_S resulted in decreased plasma levels of glutathione in H_2_S+NO groups of Control and LVH, as shown in Figure 3C. The increased concentration of glutathione resulting from the H_2_S would suppress oxidative stress [39]**,** explaining the lower plasma level of H_2_S in the H_2_S+NO groups. One can assume that in the combined treatment group there is an antagonist action between NO and H_2_S either inhibiting the synthesis or expediting the excretion of H_2_S. It is of great interest that when H_2_S concentration in the urine was determined, it showed that H_2_S excretion was increased. This finding supports the proposed theory given above that NO expedites the excretion of H_2_S from the body. This context of interaction between H_2_S and NO requires a substantial amount of further investigation to provide support, and may be a point of cross talk between the actions of H_2_S and NO.

Treatment with H_2_S+NO virtually restored the shape of the cardiac nuclei, with no evidence of fibrosis and with a few hypertrophied cells and inflammation, as shown in Figure 7A–D. Collagen content is a component of normal cardiac muscle and is also present in LV [40]. Collagen deposition and its degradation contribute importantly to vessel integrity. Administration of isoprenaline/caffeine increased cardiac collagen content in LVH-WKY; this may be due to angiotensin II which is elevated in this model and has been reported to increase the collagen deposition [41]. Collagen content was reduced in the H_2_S+NO treatment groups (LVH-H_2_S+NO) and was almost indistinguishable from normal tissue, with very few areas having collagen spots in the heart, as shown in Figure 7A–D.

It is possible to argue that the combined effect of H_2_S and NO resulted in a synergistic response which might be an additive or potentiated manner. This suggestion correlates with a previous study which reported that L-arginine upregulated the CSE/H_2_S pathway [42] with H_2_S exerting its effect on the vascular system by regulating NO [4,43]. However, the underlying mechanisms need to be elucidated as to whether the combination of both H_2_S and NO result in the formation of intermediate molecule nitrosthiol [37,43] or nitroxyl [44,45]. However, a preliminary in vitro study suggested that the combination of NaHS (H_2_S donor) and L-arginine (NO donor) administration resulted in responses which appeared to be the sum of the individual responses obtained with either L-arginine or NaHS alone [46].

The present study investigated molecular levels further to find these interactions between H_2_S and NO in physiological, pathological and pharmacological settings by quantification of CSE/H_2_S and ENOS/NO pathways in the heart (Table S7 and Figure 5A,B) and plasma to understand the exact mechanism. We reported previously that exogenous administration of the H_2_S donor upregulated the cardiac CSE/H_2_S pathway, resulting in an attenuation of the increased LV index, heart index and thickness of the myocardium [21]. We also reported that enhanced expression of endothelial nitric oxide synthase in the myocardium ameliorates the progression of left ventricular hypertrophy in L-arginine treated Wistar-Kyoto rats [4]. In the reported study [4], we have explored that exogenous administration of the NO donor has reduced the concentration of H_2_S in the plasma and promoted its excretions. The combined treatment with H_2_S+NO in the Control and LVH groups resulted in an upregulation of cardiac eNOS mRNA with a downregulation of cardiac CSE mRNA. This pattern of cardiac eNOS and CSE mRNA expression under these circumstances may be explained by the increased concentration of NO, the decreased concentration of H_2_S in plasma and the increased concentration of H_2_S in the urine. The observations of the present study are in contrast to other studies which reported that the interaction of H_2_S+NO resulted in the formation of an intermediate molecule, most probably nitrosthiol [37,47] or nitroxyl [44,45]. 

In combined treatment groups of both Control and LVH (H_2_S+NO), it was observed that the decrease in the plasma concentration of H_2_S was of the same magnitude as that observed in the LVH-NO group (as shown in Table S5) supporting the argument that in LVH, either NO antagonizes H_2_S production or the CSE/H_2_S pathway was not able to upregulate under those conditions. When CSE mRNA expression was quantified using quantitative StepOnePlus real time PCR, it was observed that CSE mRNA expression was also downregulated to a greater degree in the heart of Control-H_2_S+NO and LVH-H_2_S+NO groups. When the urine concentration of H_2_S was measured in the Control-H_2_S+NO (physiological group), urinary H_2_S excretion was almost comparable to that of the Control-H_2_S and Control-NO groups as shown in Table S5, but when urinary excretion of H_2_S was measured in the LVH-H_2_S+NO group, there was a much-enhanced excretion of H_2_S in the urine when compared to either the LVH-H_2_S or LVH-NO groups. As argued above, it is possible to conclude that when H_2_S and NO are given together under physiological conditions (Control-H_2_S+NO), there was a downregulation of CSE mRNA expression in the heart when compared to Control WKY, but it was accompanied with a comparable excretion of H_2_S from the body. On the other hand, with co-administration in a pathological setting, the LVH-H_2_S+NO group resulted in the downregulation of CSE mRNA in the heart, but also resulted in a greater urinary excretion of H_2_S compared to the LVH-H_2_S and LVH-NO groups, as shown in Table S6. In summary, co-administration of H_2_S+NO in LVH resulted in the downregulation of the CSE/H_2_S pathway; a promotion of H_2_S excretion and upregulation of the eNOS/NO pathway. Therapeutic benefits may accrue mainly due to the eNOS/NO pathway in the heart, but are unrelated to the CSE/H_2_S pathway.

In a clinical prospective point of view, combined treatment with both H_2_S+NO provides multifactorial mechanism of action and will not only arrest the progression of LVH, but will also reverse the LVH by controlling systemic parameters, improving oxidative status and maintaining the geometry of heart. 

## 5. Conclusions

Exogenous simultaneous administrations of donors of H_2_S and NO in LVH significantly ameliorate the progression of LVH by attenuating systemic hemodynamic, physical indices and increasing oxidative stress. The molecular expression findings showed that co-administration of H_2_S+NO always led to increased expression of eNOS and suppression of CSE expression in the heart, which is fully supported by the concentration measurements of H_2_S and NO in the plasma in physiological, pathological, and pharmacological in vivo settings. Exogenous simultaneous administrations of donors of H_2_S and NO in LVH reversed the morphological changes in the myocardium of LVH rats.

## Figures and Tables

**Table 1:** R-R interval, R-amplitude, QRS complex and QT interval of WKY and H_2_S+NO of Control and LVH groups on days 35.

	Parameters			
Groups	R-R Interval (sec)	R-Amplitude (mV)	QRS (sec)	QT Interval (sec)
Control WKY	0.17 ± 0.002	0.53 ± 0.01	0.017 ± 0.0001	0.070 ± 0.003
Control-H_2_S+NO	0.16 ± 0.001	0.56 ± 0.04	0.019 ± 0.0006	0.079 ± 0.002
LVH-WKY	0.20 ± 0.004 *	0.70 ± 0.02 *	0.025 ± 0.002 *	0.087 ± 0.001 *
LVH-H_2_S+NO	0.18 ± 0.002 #	0.63 ± 0.02 #	0.017 ± 0.0005 #	0.087 ± 0.003 *

The values are mean ± SEM (n = 6). *p* < 0.05. Statistical analysis was done by one-way analysis of variance followed by Bonferroni post hoc tests for all the groups. * *p* < 0.05 vs. Control WKY and # *p* < 0.05 vs. LVH-WKY on D-35.

## Data Availability

Not applicable.

## Supplementary Materials

The following supporting information can be downloaded at: https://www.mdpi.com/article/10.3390/cimb44010030/s1, Table S1: Systolic blood pressure, diastolic blood pressure, mean arterial pressure and heart rate of WKY, H2S, NO and H2S+NO of Control and LVH groups on day 35 of anesthetized rats. Table S2: Heart, LV indices and LV chamber diameter of WKY and H2S+NO of Control and LVH groups on days 35 of anesthetized rats. Table S3: R-R interval, R-amplitude, QRS complex and QT interval of WKY, H2S, NO and H2S+NO of Control and LVH groups on days 35. Table S4: Superoxide dismutase, malanodialdehyde, glutathione and total antioxidant capacity of WKY, H2S, NO and H2S+NO of Control and LVH groups on days 35. Table S5: Hydrogen sulphide (H2S) in plasma, H2S in urine, and NO in plasma of WKY, H2S, NO and H2S+NO of Control and LVH groups on days 35. Table S6: Relative quantification of heart CSE (mRNA levels) of Control WKY, Control-H2S, Control-NO, Control-H2S + NO, LVH-H2S, LVH-NO and WKY-H2S+NO. Levels of mRNA were normalized to b-actin. Table S7: Relative quantification of heart eNOS (mRNA levels) of Control WKY, Control-H2S, Control-NO, Control-H2S + NO, LVH-H2S, LVH-NO and WKY-H2S+NO. Levels of mRNA were normalized to b-actin.
