# Supplementary material for: Physiological, Pathological and Pharmacological Interactions of Hydrogen Sulphide and Nitric Oxide in the Myocardium of Rats with Left Ventricular Hypertrophy
Source: Curr Issues Mol Biol. 2022 Jan 16;44(1):433–48. doi: 10.3390/cimb44010030 (PMC8929131; doi:10.3390/cimb44010030)
Supplement: Supplementary file 1 [file cimb-44-00030-s001.zip › cimb-1515697-supplementary.pdf]

Table S1: Systolic blood pressure, diastolic blood pressure, mean arterial pressure and heart rate of WKY, H_2S, NO and H_2S+NO of Control and LVH groups on day 35 of anesthetized rats.

**Parameters**	**Groups**	**Day 35**
Systolic blood pressure (mmHg)	Control WKY	132±4
	Control- H_2S	140±7
	Control-NO	147±1
	Control- H_2S+NO	132±2
	LVH-WKY	159±5 * λ‡
	LVH-H_2S	135±2 #
	LVH-NO	133±2 #
	LVH-H_2S+NO	122±5 † #
Mean arterial pressure (mmHg)	Control WKY	119±1
	Control- H_2S	122±6
	Control-NO	128±1
	Control- H_2S+NO	117±4
	LVH-WKY	142±5 * λ‡
	LVH-H_2S	122±3 #
	LVH-NO	114±3 #
	LVH-H_2S+NO	101±5 * λ‡ # ▲

The values are mean±SEM (n= 6).P<0.05.Statistical analysis was done by one-way analysis of variance followed by Boneferroni *post hoc* test for all the groups.* P<0.05 vs. Control WKY and • P<0.05 vs. LVH-WKY on D-35.

Table S2: Heart, LV indices and LV chamber diameter of WKY and H_2S+NO of Control and LVH groups on days 35 of anesthetized rats.

**Groups**	LV index (%)	LV chamber diameter (mm)
Control WKY	0.15± 0.00	5.09±0.01
Control- H_2S	0.19±0.00 *	4.2±0.16 *
Control-NO	0.17±0.00 * λ	5.85±0.10 * λ
Control- H_2S+NO	0.20±0.00 * †	4.42±0.18 * †
LVH-WKY	0.23±0.00 * λ†‡	2.85±0.04 * †‡
LVH-H_2S	0.21±0.00 * λ† #	5.38±0.05 ‡ #
LVH-NO	0.21±0.00 * † #	4.80±0.11 λ† # ▲
LVH-H_2S+NO	0.18±0.00 * # ▲Δ	4.09±0.07 * † #▲Δ

The values are mean±SEM (n= 6).P<0.05.Statistical analysis was done by one-way analysis of variance followed by Boneferroni *post hoc* test for all the groups.* P<0.05 vs. Control WKY and • P<0.05 vs. LVH-WKY on D-35.

Table S3: R-R interval, R-amplitude, QRS complex and QT interval of WKY, H_2S, NO and H_2S+NO of Control and LVH groups on days 35.

	Parameters			
**Groups**	R-R interval (Sec)	R-amplitude (mV)	QRS (sec)	QT interval (sec)
Control WKY	0.17±0.002	0.53±0.01	0.017±0.0001	0.070±0.003
Control- H_2S	0.17±0.009	0.54±0.03	0.019±0.0006	0.076±0.003
Control-NO	0.18±0.004	0.61±0.03	0.017±0.0005	0.080±0.003
Control- H_2S+NO	0.16±0.001	0.56±0.04	0.019±0.0006	0.079±0.002
LVH-WKY	0.20±0.004 *	0.70±0.02 *	0.023±0.002 *	0.087±0.001 *
LVH-H_2S	0.18±0.002 #	0.66±0.02	0.018±0.0002 #	0.082±0.003
LVH-NO	0.16±0.001 #	0.50±0.01 #	0.017±0.0002 #	0.071±0.004 #
LVH-H_2S+NO	0.18±0.002 #	0.63±0.02 #	0.017±0.0005 #	0.087±0.003 *

The values are mean±SEM (n= 6).$P<0.05$.Statistical analysis was done by one-way analysis of variance followed by Boneferroni *post hoc* test for all the groups.* $P<0.05$ vs. Control WKY and # $P<0.05$ vs. LVH-WKY on D-35.

Table S4: Superoxide dismutase, malanodialdehyde, glutathione and total antioxidant capacity of WKY, H_2S, NO and H_2S+NO of Control and LVH groups on days 35.

	**Parameters**			
**Groups**	SOD (U/mL)	MDA (nmol/mL)	GSH (μmol/L)	T-AOC (U/mL)
Control WKY	6±0.19	21±2	542 ± 0.18	18±1
Control- H_2S	6 ±0.31	21±1	629±38	14±2
Control-NO	9±0.74 * λ	26±2	649±35	11±1 *
Control- H_2S+NO	10±0.51 * λ	22±2	359±34 * λ†	15±1 †
LVH-WKY	3±0.67 * λ†‡	34±1 * λ†‡	134±19 * λ†‡	10±0.5 * ‡
LVH-H_2S	7±0.27 ‡ #	23±1 #	731±45 ‡ #	14±0.5 * #
LVH-NO	9±0.19 * λ #	30±1 * λ‡ #	610±68 ‡ #	10±0.6 * λ‡▲
LVH-H_2S+NO	8±0.44 ‡ #	22±1 # Δ	284±12 * λ†▲Δ	14±0.5 * # Δ

The values are mean±SEM (n= 6).P<0.05.Statistical analysis was done by one-way analysis of variance followed by Boneferroni *post hoc* test for all the groups.* P<0.05 vs. Control WKY and # P<0.05 vs. LVH-WKY on D-35.

Table S5: Hydrogen sulphide (H_2S) in plasma, H_2S in urine, and NO in plasma of WKY, H_2S, NO and H_2S+NO of Control and LVH groups on days 35.

	**Parameters**		
**Groups**	H_2S in plasma (μM)	H_2S in urine (μM)	NO in plasma (μmol/L)
Control WKY	37±1	18±1	28±1
Control- H_2S	54±1 *	45±5 *	33±1 *
Control-NO	49±2 *	56±5 *	36±1 * λ
Control- H_2S+NO	19±4 * λ†	61±4 *	45±2 * λ
LVH-WKY	16±1 * λ	28±1 *	20±1 * λ†‡
LVH-H_2S	63±2 * ‡ #	45±4 * #	27±2 λ†‡ #
LVH-NO	18±1 * λ†▲	60±7 * #	34±1 * ‡ # ▲
LVH-H_2S+NO	19±3 * λ†▲	102±13 * λ† # ▲∆	35±1 * # ▲

The values are mean±SEM (n= 6).P<0.05.Statistical analysis was done by one-way analysis of variance followed by Boneferroni *post hoc* test for all the groups.* P<0.05 vs. Control WKY and # P<0.05 vs. LVH-WKY on D-35.

Table S6: Relative quantification of heart CSE (mRNA levels) of Control WKY, Control-H_2S, Control-NO, Control-H_2S + NO, LVH-H_2S, LVH-NO and WKY-H_2S+NO. Levels of mRNA were normalized to b-actin.

Sample Tissue	Groups	RQ =2^ – (ΔΔC_T)
Heart	WKY	1.00±0.00
	WKY + H_2S	1.61±0.04*
	WKY + NO	0.82±0.012* λ
	WKY- H_2S + NO	0.72±0.01* λ
	LVH-WKY	0.26±0.02* λ†‡
	LVH+ H_2S	1.48± 0.03*†‡ #
	LVH+ NO	0.26±0.006*†‡ λ▲
	LVH-H_2S+NO	0.67±0.06# λ▲Δ

Data was expressed as mean ± SEM relative to WKY for CSE mRNA (*n*= 3, triplicate samples from three separate rats in each group). Data was analyzed using the $2^{-\Delta\Delta\ CT}$ method while the differences among the groups were analyzed by one way ANOVA followed by Bonferroni post hoc test. * Represent significance in comparison with control WKY and # represents significance in comparison with LVH-WKY group.

Table S7: Relative quantification of heart eNOS (mRNA levels) of Control WKY, Control-H_2S, Control-NO, Control-H_2S + NO, LVH-H_2S, LVH-NO and WKY-H_2S+NO. Levels of mRNA were normalized to b-actin.

Sample Tissue	Groups	RQ =2^ – ($\Delta\Delta C_T$)
Heart	WKY	1.00±0.00
	WKY + H_2S	1.10±0.10
	WKY + NO	1.85±0.05* λ
	WKY- H_2S + NO	1.48±0.05* λ†
	LVH-WKY	0.66±0.006* λ†‡
	LVH+ H_2S	0.86±0.02†‡
	LVH+ NO	1.27±0.02 *†#▲
	LVH-H_2S+NO	1.32±0.06*†#▲

Data was expressed as mean ± SEM relative to WKY for CSE mRNA (*n*= 3, triplicate samples from three separate rats in each group). Data was analyzed using the $2^{-\Delta\Delta CT}$ method while the differences among the groups were analyzed by one way ANOVA followed by Bonferroni post hoc test. * Represent significance in comparison with control WKY and # represents significance in comparison with LVH-WKY group.
